# Implementation Strategies for Widespread Scaling of Effective Programs into Healthcare systems

**DOI:** 10.1093/geroni/igab046.189

**Published:** 2021-12-17

**Authors:** Jaime Hughes

**Affiliations:** Wake Forest School of Medicine, Raleigh, North Carolina, United States

## Abstract

Translation of effective evidence-based programs into practice is critical to promoting and preserving older adults’ function and independence. This presentation will provide an introduction to implementation strategies, defined as the “methods or techniques used to enhance the adoption, implementation, and sustainability of a clinical program or practice.” Some examples of implementation strategies include education and training, stakeholder engagement, patient and/or consumer involvement, adaptation, and technical assistance. Application of these implementation strategies will be illustrated using examples from local and national scale out of evidence-based health promotion programs for older adults within the VA Healthcare System. This presentation will close with guidance on how to select, track, and evaluate implementation strategies.

